# Decision criteria for the selection of analytical instruments used in clinical chemistry: I Introduction

**DOI:** 10.1155/S1463924680000077

**Published:** 1980

**Authors:** R. Haeckel

**Affiliations:** Institute for Clinical Chemistry Medical School Hannover Germany


### Decision criteria for the selection of analytical instruments used in clinical chemistry*

### I Introduction

**R. Haeckel**
*Institute for Clinical Chemistry, Medical School, Hannover, W. Germany.*

ASSOCIATE Members of the Expert Panel on Instrumentation of the International Federation of Clinical Chemistry (IFCC) suggested a need for more information on how medical laboratories should select new instruments.

It was pointed out that relatively isolated communities often find it difficult to choose rationally from the large amount of literature, which presents a bewildering array with little indication as to which instrument is most suitable for a particular application.

Therefore the Expert Panel on Instrumentation of IFCC, together with the Commission on Automation of the International Union of Pure and Applied Chemistry (IUPAC), undertook to discuss the basic problems involved during symposia held at the 3rd European Congress of Clinical Chemistry (Brighton, June 1979) and at the lst South East Asian and Pacific Congress of Clinical Biochemistry (Singapore, October 1979).

The selection of analytical instruments is a very complex problem for a clinical chemistry laboratory. The reason lies in the fact that medical, methodological, instrumental and organisational aspects must be considered at the same time. Dealing with the problem of selecting analytical instruments is part of laboratory management. Professional economists differentiate between service and economic goals (Fig.1). The service goal starts with the responsibility for the patient to be served as well as possible, whereas the economic goal is related to the responsibility towards the administration. In many cases these goals conflict with each other. The selection process usually starts if a troublesome problem is suspected. The problem will then be analysed with consideration given to the laboratory infra-structure (organisation problems), and the current state of staffing, instrumentation and mechanisation. If the analysis indicates that the purchase of a new analytical instrument could solve the problem, a decision strategy is then followed; this usually takes place more or less intuitively. The problem must be defined, and the relevant aspects of such a strategy identified. This aspect is discussed in the first paper. A list of all suitable instruments is then prepared; information produced by the manufacturers according to IFCC proposals can help to reduce the size of this list to those most appropriate for the particular application. The final choice should be made by taking into account factors under the following headings (Fig.2):

1. Non-monetary criteria, for example, analytical reliability and dependability.
2. External experiences from other laboratories which can either be obtained from evaluation reports or through enquiries from colleagues who have already used the instruments under consideration. Internal trials may be necessary to cover special circumstances.
3. The influence of new instrumentation on the infra-structure of the laboratory's organisation has been neglected. This is especially important if multi-channel analysers with internal sample splitting are to be installed. Some manufacturers have produced simulation models to determine the interaction of several factors under varying conditions.
4. Economic aspects, including cost comparison studies.

These four decision areas have been chosen for discussion in this series of papers.

**Papers for discussion prepared by members of the International Federation of Clinical Chemistry Expert Panel on Instrumentation, and the International Union of Pure and Applied Chemistry Commission on Automation, under the Chairmanship of Professor R Haeckel.*

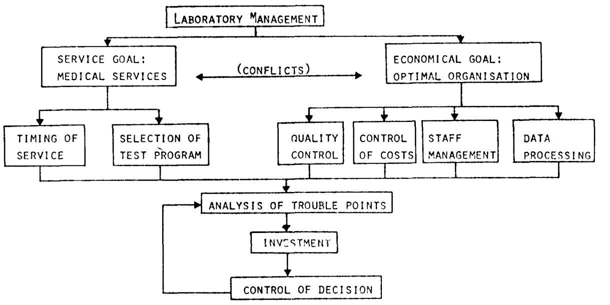

*Figure 1. Diversification of management functions in a medical laboratory*

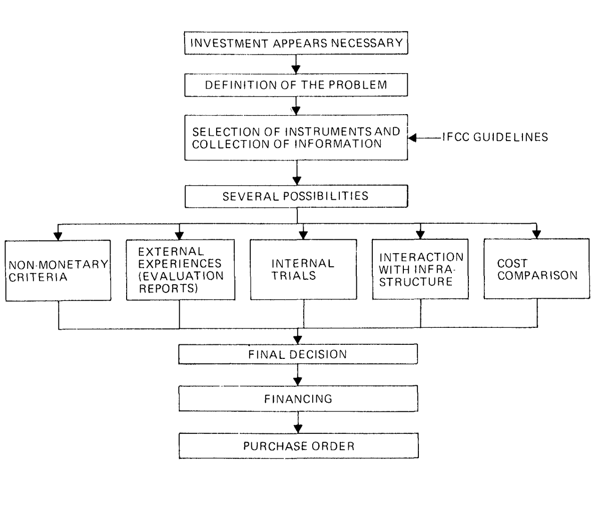

*Figure 2. Decision strategy*

